# Pilot Study on the Development and Integration of Anthropomorphic Models within the Dental Technician Curriculum

**DOI:** 10.3390/dj12040091

**Published:** 2024-04-02

**Authors:** Kristina Bliznakova, Minko Milev, Nikolay Dukov, Virginia Atanasova, Mariana Yordanova, Zhivko Bliznakov

**Affiliations:** 1 Faculty of Public Health, Medical University–Varna Prof. Dr. Paraskev Stoyanov, 9002 Varna, Bulgaria; ntdukov@mu-varna.bg (N.D.); atanasova.virginia@mu-varna.bg (V.A.); zhivko.bliznakov@mu-varna.bg (Z.B.); 2 Medical College, Medical University–Varna Prof. Dr. Paraskev Stoyanov, 9002 Varna, Bulgaria; minko.milchev.milev@mu-varna.bg (M.M.); kupenova@mu-varna.bg (M.Y.)

**Keywords:** 3D printed anthropomorphic models, dental technicians, educational phantoms, higher education

## Abstract

The effectiveness of modern medical education largely depends on the integration and utilization of digital technologies in teaching various disciplines. In this pilot usability study, we introduced 3D printed anthropomorphic dental models, specifically designed for the elective discipline “Digital and Metal-Free Techniques in Dental Technology” from the curriculum of the Dental Technician specialty in the Medical University of Varna. The evaluation focused on dental technician students’ perception of this novel learning environment, its influence on their performance, and the potential for future application of these models and related 3D technologies in their professional practice. A validated satisfaction questionnaire was distributed among 80 students, comprising the total cohort. The results indicated a high acceptance rate, with nearly 95% of participants finding the use of digitally created 3D-printed dental models beneficial. More than 90% believed that exploring digital technologies would enhance their skills. The well-trained instructor’s competence in technology use convinced students of its value, with more than 98% expressing a willingness to incorporate these technologies into their future work for improved precision in dental models. However, due to the current high cost of needed equipment, only 10% of participants may practicably introduce this novel technology into their practical work. The use of anatomically accurate 3D printed models is a valuable addition to the current dental technician curriculum in medical colleges.

## 1. Introduction

Among the key elements on which the success of modern medical education depends is the integration of advanced digital techniques into university lectures and exercises [1]. One such technique is three-dimensional (3D) printing technology, which provides opportunities for printing anatomical objects for medical education, as well as introducing 3D printers as a core tool in basic and advanced elective courses in B.Sc. and M.Sc. study programs [2,3]. In an extensive review conducted by Adnan and Xiao, which analyzed 156 research articles across six health disciplines, it was revealed that 35% of studies employed 3D digital printing tools, particularly in educational activities [4].

The digital model employed in 3D printing can be generated through computational programs, which make use of either mathematical approaches to construct the anatomical models or patient-specific data. In the latter case, data from X-ray modalities such as computed tomography (CT), as well as data from magnetic resonance imaging (MRI), or 3D ultrasound, can be utilized. In most cases, the patient-specific images are in the digital imaging and communications in medicine (DICOM) format and, after proper anonymization, can be used to extract anatomical features. On the other hand, mathematical approaches have proven successful in generating anthropomorphic models for various anatomical structures [5]. This approach is fast, flexible, and does not compromise patient privacy.

According to a study by Cercenelli et al. [6], 53% of medical students believe that 3D printing technology could enhance their confidence with medical devices. Printed realistic medical phantoms play a key role in the training of medical specialties, such as surgery [7,8], pediatrics [9], traumatology [10], as well as anatomy [11,12,13,14,15] and pathology [16]. These studies demonstrate high levels of student satisfaction, confirming that the integration of 3D printed models into the curriculum positively influences the teaching–learning outcome, such as by increasing confidence in identifying pathologies and enriching students’ learning experience.

The integration of 3D printed models into students’ education has yielded enhanced understanding of both heart diseases [17,18,19] and transthoracic echocardiography [20]. Teaching ultrasound guidance for lumbar puncture, for instance, is facilitated by a homemade spinal model [21] produced by fused deposition modeling (FDM) with nylon filaments. In the field of ophthalmology, a 3D-printed eye model was reported to significantly improve students’ study interest and study efficiency in teaching direct ophthalmoscopy to undergraduates [22,23]. Three-dimensional-printed ears have also proven invaluable in enhancing the undergraduate medical education of otolaryngology students, contributing to advancements in surgical skills and confidence among surgical residents even before engaging in real-life procedures [24]. Students in nursing are taught the skill of inserting peripheral venous catheters into veins [25] and other advanced applications [26]. 

Incorporating 3D printing technologies in the curriculum could also provide valuable knowledge and skills for students pursuing dental technology in medical colleges. Currently, there is only one reported use of a 3D model for the education of dental technicians’ students by Sheeba et al. [27]; however, there is lack of details regarding its specific incorporation into student exercises, practical applications with patients, and its potential as a foundational element for creation of a novel and updated curriculum. 

Implementing inexpensive personalized 3D printed models can be useful in surgical training for dental students and oral and maxillofacial surgery residents, as demonstrated by Werz et al. [28]. While these models received high praise for their excellence, they were found to be more suitable for surgical education rather than for training dental technician students. Furthermore, a recent study conducted by Acharya et al. [29] revealed that dental college technicians showed lower awareness and understanding of 3D printing techniques applied in dentistry when compared to their colleagues in private dental practice. The lack of knowledge is the main obstacle of implementing 3D printing technologies in dental practices [30].

This pilot usability study aims to evaluate the perception of dental technician students regarding a novel learning environment that incorporates 3D printed dental models, and its impact on their performance and future exploitation of such models and related 3D technologies in their professional work.

## 2. Materials and Methods

### 2.1. Creation of Dental Model for the Training of Dental Technicians

As a rule, in modern dentistry the transfer of soft and hard teeth-lingua from a patient to a physical model is carried out in two ways. The first one is the digital way, through direct scanning with an intraoral scanner and software generation of a 3D model, while the second one is through use of silicone and alginate model impression materials mixed in proportions—base and catalyst—according to the manufacturer’s instructions. In the first case, the generated 3D model in STL format can be further processed by software and printed by a 3D printer as a physical model with movable and immovable sectors, while in the second case, an analog impression is cast from laboratory plasters with movable sectors. 

We chose to work with a plaster model as a base for the 3D model, on which the training courses were conducted. The movable and immovable sectors were scanned using a 3Shape Redline E3 laboratory scanner, yielding results in STL format. The software of the scanner saves distinct STL files for the model with fixed teeth, removable teeth (stumps), and the restoration.

The chosen model is based on the Viola system by setting the sectors of removable tooth elements, consisting of stumps of prepared and non-prepared teeth from 1.5 to 2.4 teeth. A clear delineation of the preparation margins on two removable teeth, specifically 1.5 and 2.1, is visible. These guidelines serve as the basis for students to create a metal-free restoration. 

The 3D model for the dental technicians was produced using Formlabs Form 3 and utilized two distinct types of resin: one for the dental model (Formlabs Model V2 resin) and another for the dental stumps (Formlabs White V4 resin). Printing occurred at a layer thickness of 25 µm, with supports and mini rafts. Meanwhile, the dental stumps were printed at a layer thickness of 50 µm, also employing supports with mini rafts. The dental stumps were printed in batches of eight, where each set in a batch comprised eight stumps—equivalent to the number required to populate a printed dental model. Post-processing involved washing the dental models and stumps in Form Wash using fresh isopropyl alcohol (IPA) with a concentration of 99.5% for 10 min, followed by a post-curing phase for 60 min at 60 °C in Form Cure.

**Figure 1:**
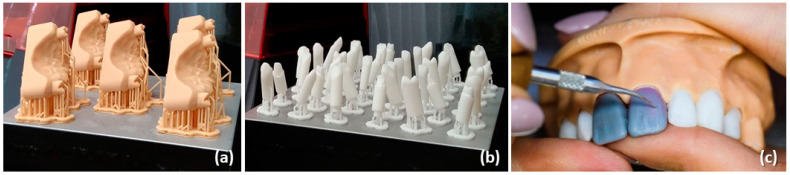
Dental model and stumps: (**a**) printed dental models, (**b**) printed dental stumps, (**c**) work on the assembled dental model.

Figure 1 shows the printed dental models and stumps, as well as the handmade wax modeling on the prepared tooth by the students for the pressing with LiSi_2_. 

**Figure 2:**
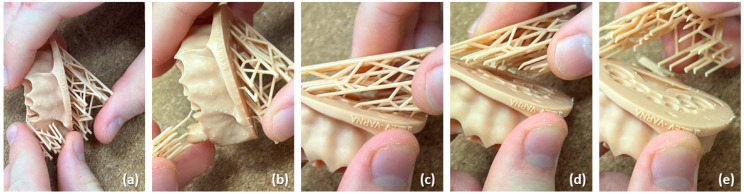
(**a**–**e**) Removal of the printing supports from the dental models.

**Figure 3:**
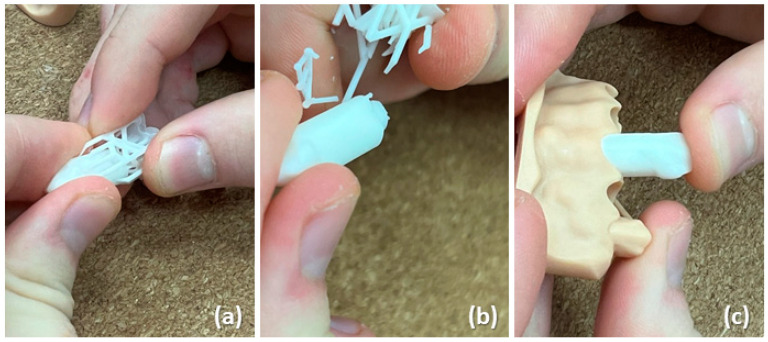
(**a**,**b**) Removal of the printing supports from the printed stumps; (**c**) insertion of the stumps into the dental model.

The digitally developed models and stumps for handmade full ceramic restoration were provided to the students, who began their work by removing the printing supports carefully (Figure 2 and Figure 3a,b). Although this step demands accuracy and manual dexterity, the removal of the printing supports was relatively easy and usually did not require additional equipment. Then, the printed stumps were inserted into the model (Figure 3c). This was highly precise work, given that each stump had a dedicated position in the model. 

The 3D printed models have exceptional accuracy and lack the hollows and imperfections that are often found in analogs handmade with plaster models [31]. Plaster models are made from a combination, most often of two plasters, each of which has different technical parameters such as difference in consistency, mixing powder with distilled water, shrinkage after hardening, and time for free setting of an analog silicone impression. The two types of plaster are cast separately, which involves a wait time recommended by the plaster manufacturer. The combination of these waiting times can be higher than the 3D printing time of the same models. Furthermore, the price ranges of hard plasters for casting the working parts are usually 4th and 5th grade, close to or even more expensive than the price of the resin used to print models using digital technology.

**Figure 4:**
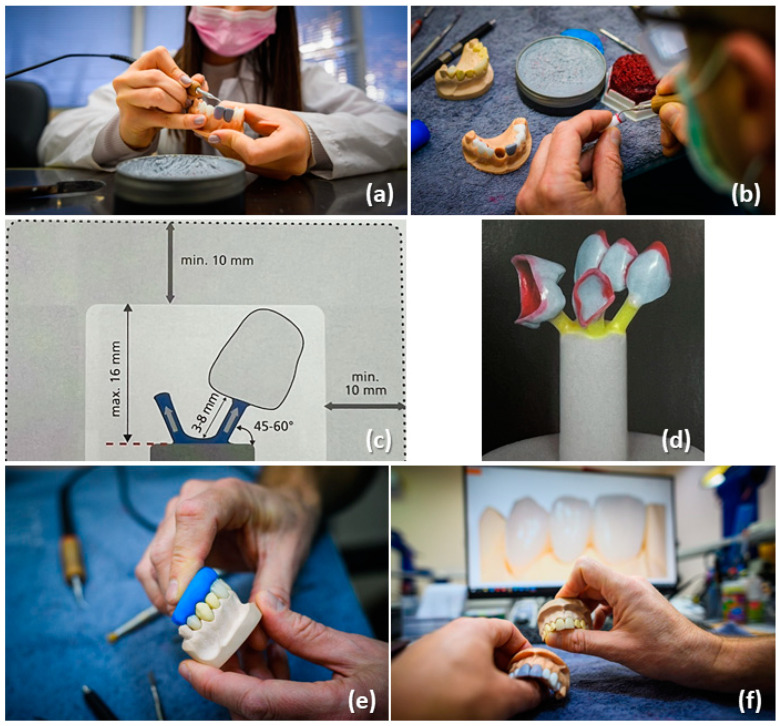
Basic actions, implemented by the students in their training: (**a**) wax modeling, (**b**) refining the cervical area, (**c**) utilizing a template from the instructions, (**d**) securing the wax crown onto a specialized form, and (**e**,**f**) completing the restoration with LiSi_2_ and a glaze material.

Figure 4 illustrates the main activities undertaken by students while performing handmade wax modeling on a prepared tooth. The process began with wax modeling (Figure 4a), followed by refining the cervical area (Figure 4b). Next, students adhered to the guidelines provided in the manufacturer’s instructions (Figure 4c), then secured the wax crown onto a specialized form (Figure 4d). The final step involved completing the restoration using LiSi_2_ and a glaze material (Figure 4e,f). These steps were spread across 29 h over five consecutive weeks to guarantee comprehensive completion.

Each dental model was estimated to have a cost of EUR 3, while a set of dental stumps needed for populating the dental model costs approximately EUR 2. In order to produce these models, our department invested in an SLA printer, which cost EUR 4300. For the manufacturing of the described models, we used two types of resins, Model V2 resin and White V4 resin, both of which cost EUR 170 per cartridge (a cartridge contains one liter of resin). Furthermore, we used two resin tanks for the two resins, each at a cost of about EUR 170. Finally, we used post-processing equipment for washing the models and post-curing with a cost of about EUR 680 and EUR 870, respectively. The washing equipment needed to be filled with about 9 liters of IPA, which could be used multiple times until requiring change.

### 2.2. Student Assessment and Statistical Analysis

Second-year college students enrolled in the elective discipline “Digital and Metal-Free Techniques in Dental Technology” comprised the participants in this study. This discipline offers specialized education for dental technicians and is uniquely incorporated in the curriculum of the Medical College of Varna, making it distinct from the offerings at the other two medical colleges in Bulgaria. Accordingly, the participants encompassed the entire cohort.

The study was approved by the institution’s Research Ethics Committee prior to its start (approval number: 123/15.12.2021), and informed consent was obtained from all individual participants. The voluntarily undertaken and anonymous custom-made questionnaire, organized into five sections encompassing a total of 13 questions (Table 1, including an open-ended question, Question 2), was administered upon the end of the entire elective course during the winter semesters of 2021–2022 and 2022–2023. 

We conducted a small-scale trial among 20 randomly selected participants from the first academic year (2021–2022). The idea was to identify any issues with question wording, response options, and instructions. Based on the feedback provided by the participants, we adjusted the questionnaire.

The study involved 80 participants, who comprised the entire cohort. The statistical analysis was performed using RStudio package. The study used categorical variables and the results are presented as numbers and percentages. To study the relationship between any two variables, a Spearman’s correlation analysis was applied, which is presented in details in Appendix A section. To perform a deeper analysis, we employed the chi-squared test specifically on the significant correlations, refining our focus to statistically relevant associations. These are also reported in Appendix A section. 

## 3. Results

**Figure 5:**
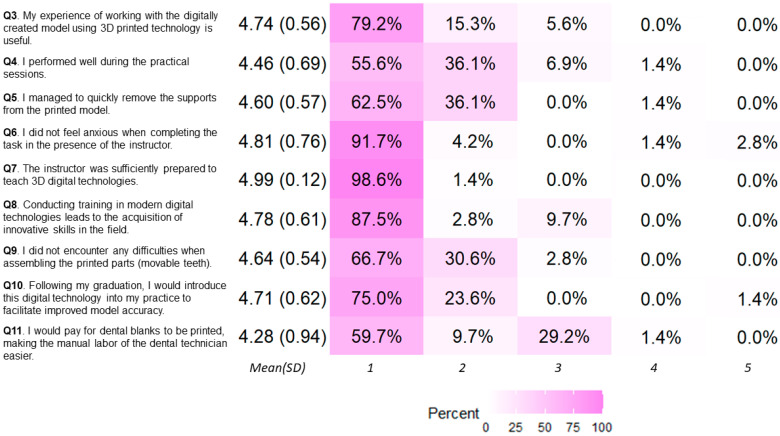
The responses of the study. The direction of positive to negative connotation is shown from 1 to 5 (“strongly agree” being 1, while “strongly disagree“ is 5).

**Figure A1:**
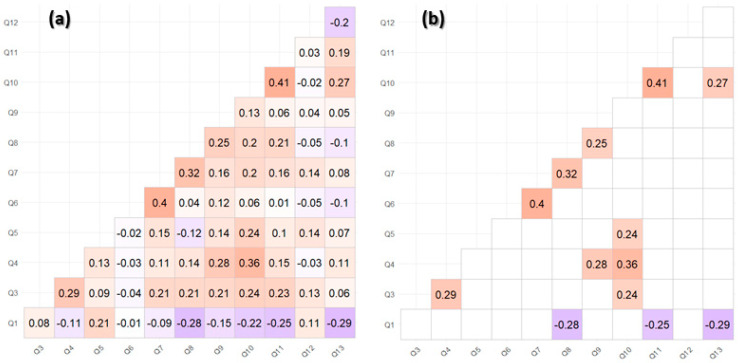
Correlation analysis: (**a**) the complete correlation matrix, (**b**) the correlation matrix with data omitted for non-significant correlations.

A total of 72 students out of 80 responded to the questionnaire, 43 women (59.72%) and 29 men (40.28%). Non-binary gender was not considered. Students′ age was divided in two age groups: 48 students had an age within the interval 18–24, while the remaining 24 students were older than 24 years. Figure 5 displays the Likert summary of the questions, excluding Question 2, which was an open-ended question, while Table 2 summarizes the results for each question with respect to the participants’ gender and age. Finally, the Appendix A section presents the item correlation matrix (Figure A1), highlighting statistically significant results with 0.01 < *p* < 0.05.

Almost one-third of the dental technician students reported an absence of previous experience during their training, which was due to the lack of digital technologies in their current curriculum. However, this type of training is in high demand, due to the continuous development of novel dental technologies [32]. Among the students in this cohort who reported prior exposure to digital technologies, females and younger participants demonstrated greater familiarity with these technologies compared to their male and older peers. While there was no observed correlation between age of participants and their gender, it is worth saying that a negative weak correlation between age of participants and previous experience existed.

The results from the questions corresponding to Experience, evaluation, and self-evaluation were positive, as nearly 95% of the participants expressed that working with digitally created 3D-printed dental models was useful (Figure 5), with more than 90% agreement within each subgroup, categorized by gender and sex (Table 2). A majority of participants (more than 90%) successfully completed the assigned practical tasks; students reported enhanced performance particularly in removing the supports from the 3D printed model, and assembling the printed parts (movable teeth) without difficulty, as shown from the responses to Q9 (Figure 5, Table 2).

The responses of the students in respect to the open question (Q2): “What do you think the advantages of using digital technologies over analog ones are?” are summarized in Table 3. Some students provided more than one point of feedback. Overall, the dental technician students reported improved accuracy and reduced manufacturing time for dental models, which allowed them to be more efficient in the laboratory and quickly produce final dental restorations. The students also identified that when working with analog models, i.e., gypsum models (during mandatory courses), there was a need for more time for the creation of full ceramic crowns and veneers, compared to working with the digitally based models used in this study. Finally, an advantage of using 3D models was that the initial printed models were the same for all students, providing a more objective evaluation of their skills.

The experience of using new technologies during this training convinced future dental technicians (more than 98% of participants) to incorporate these technologies into their routine work, with the aim of enhancing the precision of their dental models (r_s_ = 0.36, *p* < 0.002, Table A1). Results from the chi-square test of independence demonstrated that for dental technicians, the knowledge and experience gained during training plays an important role in the future adoption of digital technologies in their practice, with the aim of creating more precise dental models (χ^2^ = 12.56, *p* < 0.02). Further, dental technicians students believed that digital technologies result in acquisition of innovative skills in the field and therefore enhance their profession (more than 90% of all participants). 

In respect to the two questions about the instructor’s ability to teach the subject and help with exercises, 96% of participants expressed positive feedback. The instructor’s presence during exercises had a positive impact, helping students to perform their work without anxiety, as shown in Table A2 (χ^2^ = 72, *p* < 0.001). There was a functional correlation between “The instructor was sufficiently prepared to teach 3D digital technologies” and “Conducting training in modern digital technologies leads to the acquisition of innovative skills in the field”, shown in Table A2, which suggests that highly prepared and knowledgeable specialists are needed for students to acquire and use new technologies in their professional work. The better prepared the instructor, the more willing the students are to implement the new technologies in their practical work (χ^2^ = 9.42, *p* < 0.03; r_s_ = 0.32, *p* < 0.0056). However, due to the current economic and social development of the country, and the high cost of these technologies (investment in the printer, materials, maintenance, etc.), only 10% of the students felt they may be able to introduce the novel technology in their practical work.

The detailed correlation analysis presented in Appendix A shows that more than 97% of future dental technicians surveyed would prefer to use 3D printing technology to facilitate their work (r_s_ = 0.41, *p* < 0.0004, Table A3). There was statistical significance in the correlation between Q10 “After graduation, I would introduce in my practice this digital technology to create the most accurate model” and Q11 “I would pay for dental blanks to be printed, making the manual labor of the dental technician easier”, as shown in Table A3 (χ^2^ = 14.10, *p* < 0.03), in respect to the creation of more accurate dental models. Furthermore, students who performed well during the exercises assessed positively the usefulness of introducing this new approach of teaching the discipline of “Digital and Metal-Free Techniques” in dental technology. They conveyed their opinions on the value of acquiring new knowledge and skills, with more than 83% of participants expressing favorable sentiments. Students who successfully completed the exercise and managed the whole process well would pay for dental blanks to be printed, making the manual labor of the dental technician easier (over 66% of participants). However, this correlation was weak, as only about 17% of the students who successfully performed the exercise would be prone to such action.

## 4. Discussion

Three-dimensional printing is playing an increasingly important role in education, particularly in the field of anatomy. Using 3D printed anatomical models as an educational tool has been shown to improve students’ understanding of complex anatomical structures [33,34]. These methods are also highly valued in college education, where they help students to be integrated into the clinical environments and develop their creativity when handling complex cases. Despite conducting searches in Scopus and PubMed databases using keywords such as “3D printing”, “dental technician education”, and “additive manufacturing in dentistry”, no relevant studies on this topic were identified. The Delphi study of Logest et al. [30] reveals that one of the obstacles of introducing additive manufacturing in dental practice is the lack of knowledge of additive manufacturing. These findings further emphasize the necessity for innovative educational approaches in training dental technicians.

This study involved the participation of students from the specialty dental technician program of the Medical University of Varna. The negative weak correlation between age of participants and previous experience demonstrated in Figure A1 may be attributed to the fact that in recent years, technological improvements have led to the transformation of schools and university education, enhancing the experience of students and schoolmates [35], and younger peers have fully benefited. This result will be taken into account in the developing of educational materials and the suitable choice for its presentation.

Digital technologies have made their way into the practice of private laboratories; however, this is not the case for educational activities of dental technicians. A study of Acharya et al. [29] revealed that none of the dental technicians participating in the questionnaire agreed that 3D printing technology could be utilized as an educational tool in dentistry. However, their findings indicated that nearly 99% of dental technicians expressed an interest in incorporating 3D printing techniques into their practice. They believed that embracing digital technologies would not only enhance their professional skills but also lead to the acquisition of innovative capabilities in the field. Our study strongly supports these findings, reinforcing the idea that there is a distinct need for educational initiatives in this area.

Further, the opinions of dental technician students about the improved accuracy and reduced manufacturing time for dental models were consistent with the findings of Son et al. [36], who highlighted the advantages of 3D printing over traditional technologies in crown production, specifically in terms of achieving higher surface precision and uniformity. Similarly, Nemeth et al. [37] underlined that traditional analog approaches can be prone to errors due to inherent inaccuracies in various materials and human error. In this study, we used SLA technique to print out the dental models. A study by Nemeth et al. [37] and Grassia et al. [38] showed that the SLA technique is one of the most accurate technologies for generating full-arch dental models, excelling in both trueness and precision. This observation is also supported by the comprehensive review of Etemad-Shahidi et al. [39]. These findings suggest that the used 3D printing technology is appropriate for producing three-dimensional realistic dental models suitable for educational purposes. Future work is related to the creation of a local library dataset comprising dental models with different cases; this database will be used for training and educational purposes, preparing students for various cases tailored to individual patients. 

This study revealed that more than 97% of future dental technicians expressed a preference for integrating 3D printing technology into their workflow. Nevertheless, the initial investment required for adopting this innovative technology may pose a challenge. A study by Loges et al. [30] also identified the high cost of investing of equipment, including the necessary intraoral scanner, CAD software, and 3D printer mainly for large supply chains, while it may be beneficial and cost-effective in small production quantities. 

To successfully implement new technologies in education, the instructor must be well-educated and possess the necessary knowledge and experience [40]. The results of this study also support this, as the instructor’s competence was evident from the descriptive statistics in Figure 5, which in this study was partially due to the instructor’s involvement in research projects related to the development and use of new 3D printing technologies. 

The benefits of using digital technologies in medicine are well known, but there are also ethical issues concerns, which must be taken into account when digital technologies are used in the education of medical students [41,42]. These issues are presented by the instructor to the students, so they learn at very initial stage of their education the ethical principles in research and practical clinical work and can apply them after their graduation. The use of anthropomorphic dental models for trainings of dental technicians reveals an advantage in this aspect. In addition, these models may be more accessible to learners than cadaveric specimens [43], although one study [44] demonstrated no significant difference in students’ performance in anatomy learning when comparing groups using cadaveric specimens and 3D printed models of upper-limb musculoskeletal anatomy. 

Finally, the approach used by the instructor was in line with the current national strategy for modernization of higher education as well as also being in line with the digital education action plan [45], where the development of digital competences and skills is in focus. Acquisition of these skills by future dental technicians will result in opportunities for their career development as well as improved quality of healthcare services. 

## 5. Conclusions

The significantly positive responses, with nearly 95% agreement, regarding the usefulness of working with digitally created 3D printed dental models, along with the high success rate (more than 90%) in completing practical tasks, demonstrated a strong endorsement of the efficacy and performance benefits of this new learning environment among the participants. Advantages of introducing new technologies were well understood by the students, as these related to both reduced time and improved quality in completing specific procedures for the specialty. Furthermore, the positive impact of instructor guidance during exercises underscored its beneficial influence on all students. Overall, the exposure to new technologies in this training appears to convincingly inspire future dental technicians to incorporate these advancements into their routine work.

## Figures and Tables

**Table 1:** A questionnaire for dental technician students.

I	Previous experience.
1	Do you have any experience so far with the use of modern digital technologies during your studies? ○ Yes. ○ No.
II	Experience, evaluation and self-evaluation.
2	What do you think the advantages of using digital technologies over analog ones (e.g., gypsum models) are?
3	My experience of working with the digitally created model using 3D printed technology is useful. ○ Strongly agree ○ Agree. ○ Neither agree nor disagree ○ Disagree ○ Strongly disagree
4	I performed well during the practical sessions. ○ Strongly agree ○ Agree. ○ Neither agree nor disagree ○ Disagree ○ Strongly disagree
5	I managed to quickly remove the supports from the printed model. ○ Strongly agree ○ Agree. ○ Neither agree nor disagree ○ Disagree ○ Strongly disagree
III	Instructor
6	I did not feel anxious when completing the task in the presence of the instructor. ○ Strongly agree ○ Agree. ○ Neither agree nor disagree ○ Disagree ○ Strongly disagree
7	The instructor was sufficiently prepared to teach 3D digital technologies. ○ Strongly agree ○ Agree. ○ Neither agree nor disagree ○ Disagree ○ Strongly disagree
IV	Use of digital technology in professional work of dental technicians
8	Conducting training in modern digital technologies leads to the acquisition of innovative skills in the field. ○ Strongly agree ○ Agree. ○ Neither agree nor disagree ○ Disagree ○ Strongly disagree
9	I did not encounter any difficulties when assembling the printed parts (movable teeth). ○ Strongly agree ○ Agree. ○ Neither agree nor disagree ○ Disagree ○ Strongly disagree
10	Following my graduation, I would introduce this digital technology into my practice to facilitate improved model accuracy. ○ Strongly agree ○ Agree. ○ Neither agree nor disagree ○ Disagree ○ Strongly disagree
11	I would pay for dental blanks to be printed, making the manual labor of the dental technician easier. ○ Strongly agree ○ Agree. ○ Neither agree nor disagree ○ Disagree ○ Strongly disagree
V	Personal details
12	Your gender:
13	Age:

**Table 2:** The responses of the study regarding variations in questionnaire replies based on gender and age.

Question	Female		Male		χ^2^ Test	18–24		>24		χ^2^ Test
	N	%	N	%		N	%	N	%	
Q1. Do you have any experience so far with the use of modern digital technologies during your studies?	43		29		0.52	48		24		4.79
No	13	30%	12	41%		12	25%	13	54%	(*p* < 0.05)
Yes	30	70%	17	59%		36	75%	11	46%	
Q3. My experience of working with the digitally created model using 3D printed technology was useful.					1.37					1.72
Strongly Agree	36	84%	21	72%		37	77%	20	83%	
Agree	5	12%	6	21%		9	19%	2	8%	
Neither agree nor disagree	2	5%	2	7%		2	4%	2	8%	
Q4. I performed well during the practical sessions.					5.23					1.29
Strongly Agree	24	56%	16	55%		25	52%	15	62%	
Agree	14	33%	12	41%		18	38%	8	33%	
Neither agree nor disagree	5	12%				4	8%	1	4%	
Disagree			1	3%		1	2%	0	0%	
Q5. I managed to quickly remove the supports from the printed model.					2.27					0.68
Strongly Agree	29	67%	16	55%		29	60%	16	67%	
Agree	14	33%	12	41%		18	38%	8	33%	
Disagree			1	3%		1	2%			
Q6. I did not feel anxious when completing the task in the presence of the instructor.					2.90					2.32
Strongly Agree	39	91%	27	93%		45	94%	21	88%	
Agree	2	5%	1	3%		1	2%	2	8%	
Disagree			1	3%		1	2%			
Strongly disagree	2	5%				1	2%	1	4%	
Q7. The instructor was sufficiently prepared to teach 3D digital technologies.					0.04					0
Strongly agree	43	100%	28	97%		47	98%	24	100%	
Agree			1	3%		1	2%			
Q8. Conducting training in modern digital technologies leads to the acquisition of innovative skills in the field.					1.39					2.86
Strongly Agree	37	89%	26	90%		43	90%	20	83%	
Agree	2	5%				2	4%			
Neither agree nor disagree	4	9%	3	10%		3	6%	4	17%	
Q9. I did not encounter any difficulties when assembling the printed parts (movable teeth).					3.11					0.71
Strongly Agree	29	67%	19	67%		31	65%	17	71%	
Agree	14	33%	8	28%		16	33%	6	25%	
Neither agree nor disagree			2	7%		1	2%	1	4%	
Q10. Following my graduation, I would introduce this digital technology into my practice to facilitate improved model accuracy.					0.68					5.39
Strongly Agree	32	74%	22	76%		32	67%	22	92%	
Agree	10	23%	7	24%		15	31%	2	8%	
Strongly disagree	1	2%				1	2%			
Q11. I would pay for dental blanks to be printed, making the manual labor of the dental technician easier.					1.55					5.62
Strongly Agree	26	60%	17	59%		25	52%	18	75%	
Agree	5	12%	2	7%		7	15%			
Neither agree nor disagree	11	26%	10	34%		15	31%	6	25%	
Disagree	1	2%				1	2%			
Q12. Gender										2.09
Female						32	67%	11	46%	
Male						16	33%	13	54%	
Q13. Age					2.09					
18–24	32	74%	16	55%						
>24	11	26%	13	45%						

**Table 3:** The responses of the study in respect to the open question (Q2): “What do you think the advantages of using digital technologies over analog ones are?” The frequency was computed by normalizing the number of occurrences for a given response to the total number of answers, and subsequently expressed in percentage.

Q2. What Do You Think Are the Advantages of Using Digital Technologies over Analog Ones (e.g., Gypsum Models) Are?	Frequency
It achieves greater manufacturing accuracy of patterns, better quality, and lack of human factors such as fatigue in manufacturing.	49%
Saving time that can be used for other activities in the laboratory.	39%
During the training process the models are the same for everyone; that is, everyone models and works on the same model.	4%
Easier work.	4%
In case of model damage, we can always run the same new one, because it is saved as a file on the computer.	2%
Fewer mistakes.	1%
Cleaner.	1%

## Data Availability

The content generated and analyzed in this study is available from the corresponding author on request.

## Appendix A

We performed all statistical analyses using the RStudio package. The study used categorical variables and the results are presented as numbers and percentages. To study the relationship between any two variables, we applied Spearman’s correlation analysis. For items with a significant correlation, we performed a χ^2^ test. We considered *p*-values less than 0.05 to be statistically significant. Figure A1a shows the complete item correlation matrix. Most of the results were not statistically significant for the Spearman correlation analysis; therefore, Figure A1b shows the correlation matrix for the statistically significant results for *p* < 0.05 and *p* < 0.01. The chi-squared test results from the questionnaire are summarized in Table A1, Table A2 and Table A3 only for the statistically significant results (*p* < 0.05).

**Table A1:** Summarized results of χ^2^ test and correlation: The influence of the practical sessions on the introduction of the new technology in professional work.

(Q10) Following My Graduation, I Would Introduce This Digital Technology into My Practice to Facilitate Improved Model Accuracy.	(Q4) I Performed Well during the Practical Sessions.								Test χ^2^ (*p*-Value)	Corr. Coeff. (*p*-Value)
	Agree		Disagree		Neither Agree Nor Disagree		Strongly Agree			
	N	%	N	%	N	%	N	%		
Strongly agree	14	54%	0	0	3	40%	36	10%	12.56 (0.02)	0.36 (0.002)
Agree	11	42%	1	100%	2	60%	4	90%		
Disagree	1	4%	0		0	0%	0	0%		
Total	26		1		5		40			

**Table A2:** Summarized results of χ^2^ test and correlation: The influence of the instructor on the student’s ability to perform the exercises smoothly and acquiring of new knowledge and skills.

	(Q7) The Instructor Is Sufficiently Prepared to Teach 3D Digital Technologies.				Test χ^2^ (*p*-Value)	Corr. Coeff. (*p*-Value)
	Agree		Strongly Agree			
	N	%	N	%		
(Q6) I did not feel anxious when completing the task in the presence of the instructor.					72 (0.001)	0.40 (0.0005)
Strongly agree	0	0%	66	93%		
Agree	0	0%	3	4%		
Disagree	1	100%	0	0%		
Strongly disagree	0	0%	2	3%		
Total	1		71			
(Q8) Conducting training in modern digital technologies leads to the acquisition of innovative skills in the field.					9.42 (0.03)	0.32 (0.0056)
Strongly agree	0	0%	63	89%		
Agree	0	0%	2	3%		
Neither agree nor disagree	1	100%	6	8%		
Total	11		71			

**Table A3:** Summarized results of χ^2^ test and correlation: The influence of the need for precise model and time required for its production.

(Q11) I Would Pay for Dental Blanks to Be Printed, Making the Manual Labor of the Dental Technician Easier.	(Q10) After Graduation, I Would Introduce in My Practice This Digital Technology to Create the Most Accurate Model.						Test χ^2^ (*p*-Value)	Corr. Coeff (*p*-Value)
	Agree		Strongly Agree		Strongly Disagree			
	N	%	N	%	N	%		
Strongly agree	5	29%	38	70%	0	0%	14.10 (0.03)	0.41 (0.0004)
Agree	2	12%	5	9%	0	0%		
Neither agree nor disagree	9	53%	11	21%	1	100%		
Disagree	1	6%	0	0%	0	0%		
Total	17		54		1			
